# Setting the Pace: New Insights into Central Pattern Generator Interactions in Box Jellyfish Swimming

**DOI:** 10.1371/journal.pone.0027201

**Published:** 2011-11-02

**Authors:** Anna Lisa Stöckl, Ronald Petie, Dan-Eric Nilsson

**Affiliations:** Vision Group, Department of Biology, Lund University, Lund, Sweden; Harvard University, United States of America

## Abstract

Central Pattern Generators (CPGs) produce rhythmic behaviour across all animal phyla. Cnidarians, which have a radially symmetric nervous system and pacemaker centres in multiples of four, provide an interesting comparison to bilaterian animals for studying the coordination between CPGs. The box jellyfish *Tripedalia cystophora* is remarkable among cnidarians due to its most elaborate visual system. Together with their ability to actively swim and steer, they use their visual system for multiple types of behaviour. The four swim CPGs are directly regulated by visual input. In this study, we addressed the question of how the four pacemaker centres of this radial symmetric cnidarian interact. We based our investigation on high speed camera observations of the timing of swim pulses of tethered animals (*Tripedalia cystophora*) with one or four rhopalia, under different simple light regimes. Additionally, we developed a numerical model of pacemaker interactions based on the inter pulse interval distribution of animals with one rhopalium. We showed that the model with fully resetting coupling and hyperpolarization of the pacemaker potential below baseline fitted the experimental data best. Moreover, the model of four swim pacemakers alone underscored the proportion of long inter pulse intervals (IPIs) considerably. Both in terms of the long IPIs as well as the overall swim pulse distribution, the simulation of two CPGs provided a better fit than that of four. We therefore suggest additional sources of pacemaker control than just visual input. We provide guidelines for future research on the physiological linkage of the cubozoan CPGs and show the insight from bilaterian CPG research, which show that pacemakers have to be studied in their bodily and nervous environment to capture all their functional features, are also manifest in cnidarians.

## Introduction

Central Pattern Generators (CPGs) produce rhythmic behaviors across all animal phyla [1], [2], [3], [4]. Recent work has shown that CPGs are best studied in their bodily and nervous environment to understand their characteristics and function properly [5], [6]. Due to their fundamentally different body plan and nervous system organization, cnidarian CPGs provide an interesting comparison to those of bilaterians. Cnidarians do not possess a single integrative center, like the bilaterian brain, but typically have integrative centers, arranged in multiples of four in a radially symmetric system. Medusae (jellyfish) of Cubozoan cnidarians (box jellyfish) have one such centre in each of their quadrants [7]. Information is transmitted across their body by diffuse bipolar nerve nets and the ring nerve, a central nerve like structure containing several specialized conduction pathways [7], [8], [9], [10], [11].

**Figure 1:**
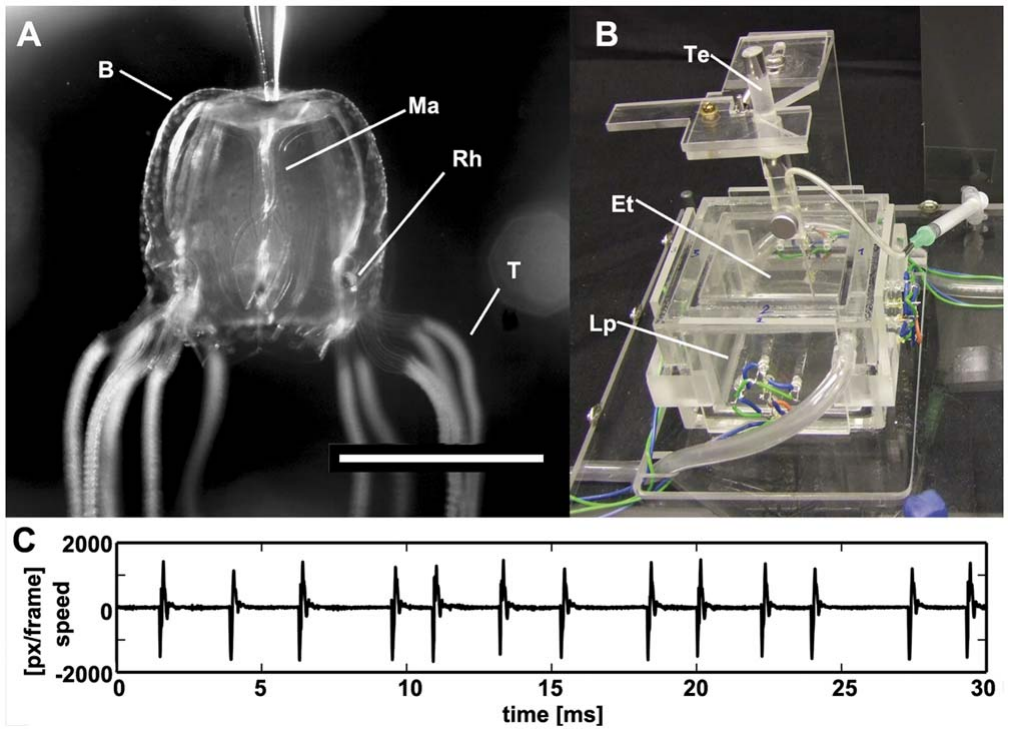
*Tripedalia cystophora*, experimental setup, and example traces of swim pulses. A tethered *Tripedalia cystophora* medusa (A), with rhopalia (Rh), manubrium (Ma), bell (B) and tentacles (T) indicated. Animals where tethered in the experimental setup (B) with suction at the top of its bell (Te) in an experimental tank (Et) with inner dimensions of 5×5×5 cm. Light stimulation was supplied by LED panels (Lp) attached to the outside of the chamber. A high speed camera looking up through the experimental tank was recording the pulsing animal. The swim pulses were extracted by tracking the speed of one rhopalium of the animal. An example trace (C) shows pulses of an animal with one rhopalium in the *light* condition. Scale bar: 0.5 cm.

The box jellyfish *Tripedalia cystophora* (Fig.1, A) is remarkable among cnidarians due to its elaborate visual system [12], which, together with the ability to actively swim and steer is used for controlling several different behaviors. Among these are obstacle avoidance, and light-shaft attraction to stay close to the prey - small copepods, which gather in the beams of light built by the leaves of mangroves in Caribbean mangrove swamps [13], [14], [15]. The visual system of all box jellyfish is located at the four rhopalia, which each contain six eyes of four different types with different optical properties and output signals through the epithelial nerve of the stalk [12], [16], [17]. Most prominent is the pair of lens eyes, which are morphologically similar to the camera type eyes of cephalopods and vertebrates [12], [18]. The central pattern generators, which have not been identified on a cellular level yet, were coarsely located to the top part of the rhopalium by ablation experiments [19], [20]. They are directly regulated by visual input [16] and control the swim pulses in a one-to-one manner with the spikes they generate [21], [22], [23]. Excision of all four rhopalia leaves the animals unable to produce spontaneous swim contractions.[22], [23], [24].

Although the electrophysiological signals of isolated pacemakers are well studied, little is known about how they interact to set the overall swim speed in intact medusae. Networks of multiple pacemakers increase the regularity of the rhythmic output of the swimming system and the absolute swim frequency as compared to single pacemakers [25]–[26]. Here, we make the first detailed analysis of swim pulses of *T. cystophora* medusae in different light conditions, with one and four rhopalia.

Since the beginning of research on the jellyfish swimming system, the standard hypothesis of the interaction of the CPGs suggests fully resetting links between them, where the fastest spiking CPG resets all other pacemakers to baseline [27]–[29]. A recent modeling study challenges this idea and proposes a semi-resetting mode of interaction between the pacemakers of the cubomedusa *Carybdea marsupialis* [26]. In order to approach the question of pacemaker interaction in more detail, we developed a numerical model which built on existing models in terms of using the swim pulse information of animals with a single pacemaker center (and correspondingly a single rhopalium) as a basis for modeling multiple pacemakers in a network [26], [28], [29]. Additional to implementing either independent pacemakers or a fully resetting interaction, we took into account different strengths of coupling between the pacemakers and modelled three different strategies of coupling.

Based on the modeling results, we propose that the CPGs are coupled via resetting links, which hyperpolarize the pacemakers below their baseline potential, confirming and extending earlier theoretical approaches to this question [27] [25] and contrasting results from a different cubozoan species [26]. We also show that a simple resetting interaction of four swim pacemakers could not account for the proportion of long inter pulse intervals (IPIs) of the animals, and suggest additional mechanisms controlling the pulse rate in box jellyfish. Our results provide guidelines for future research on the physiological links of cubozoan CPGs and show that, just as in bilaterians [5], it is necessary to study pacemakers in their neural and physiological environment in the body to become aware of all aspects of their function.

## Materials and Methods

Animals of the box jellyfish species *T. cystophora* of 3–6 mm in bell diameter were taken from cultures kept at Lund University, Sweden, and Copenhagen University, Denmark.

### Experiments

The experimental setup was a custom built double Perspex cube with an inner diameter of 5×5×5 centimeters (Fig.1, B) [30]. All experiments were performed in seawater of 25‰ salinity at 27°C, taken from the rearing tanks of the animals. In order to hold the medusa in place in the setup, it was gently attached by the apex of the bell a using suction pipette (Fig.1, B). To facilitate mounting, the animal was anesthetized by a 1∶1 mixture of 0.37 M magnesium chloride and seawater. The animal was allowed to recover for 10 minutes before the start of experiments, which restored its pulse rate to the original values [30]. All experiments were performed in the dark.

The tethered animal was visually stimulated by four panels carrying four inward facing blue-green LEDs each. The light intensity with all four panels switched on was 97.69 cd/m^2^, and 0.04 cd/m^2^ with all four panels switched off. Light intensities were measured with a photometer (Universal photometer/radiometer Model S3, B. Hagner AB, Solna, Sweden). Image sequences were shot using infrared light (IR LED) at 150 frames per second with a high-speed camera (MotionBlitz EoSens mini1, Mikrotron GmbH, Unterschleißheim, Germany). The pulse timing of the animals was obtained by tracking the spatial coordinates of one of the rhopalia (Fig.1, C) using the Mtrack2 plugin for ImageJ (http://rsb.info.nih.gov/ij/) written by Nico Stuurman (http://valelab.ucsf.edu/~nico/IJplugins/). Further analysis was done in MATLAB (MathWorks, Natick, Massachusetts, USA).

The recording protocol included four different light conditions: *light-ON* and *light-OFF*, in which recording started with switching all panels on or off, respectively, as well as constant *light* (all panels on), constant *dark* (all panels switched off). The constant light conditions were recorded after 5 minutes adaptation time. One recording run was 30 s. The first set of recordings comprising of repetitions of the four different light conditions was carried out on intact animals with four rhopalia. The second set of recordings was conducted after removing three rhopalia with fine scissors while the animal was anesthetized as explained above.

### Model

**Figure 2:**
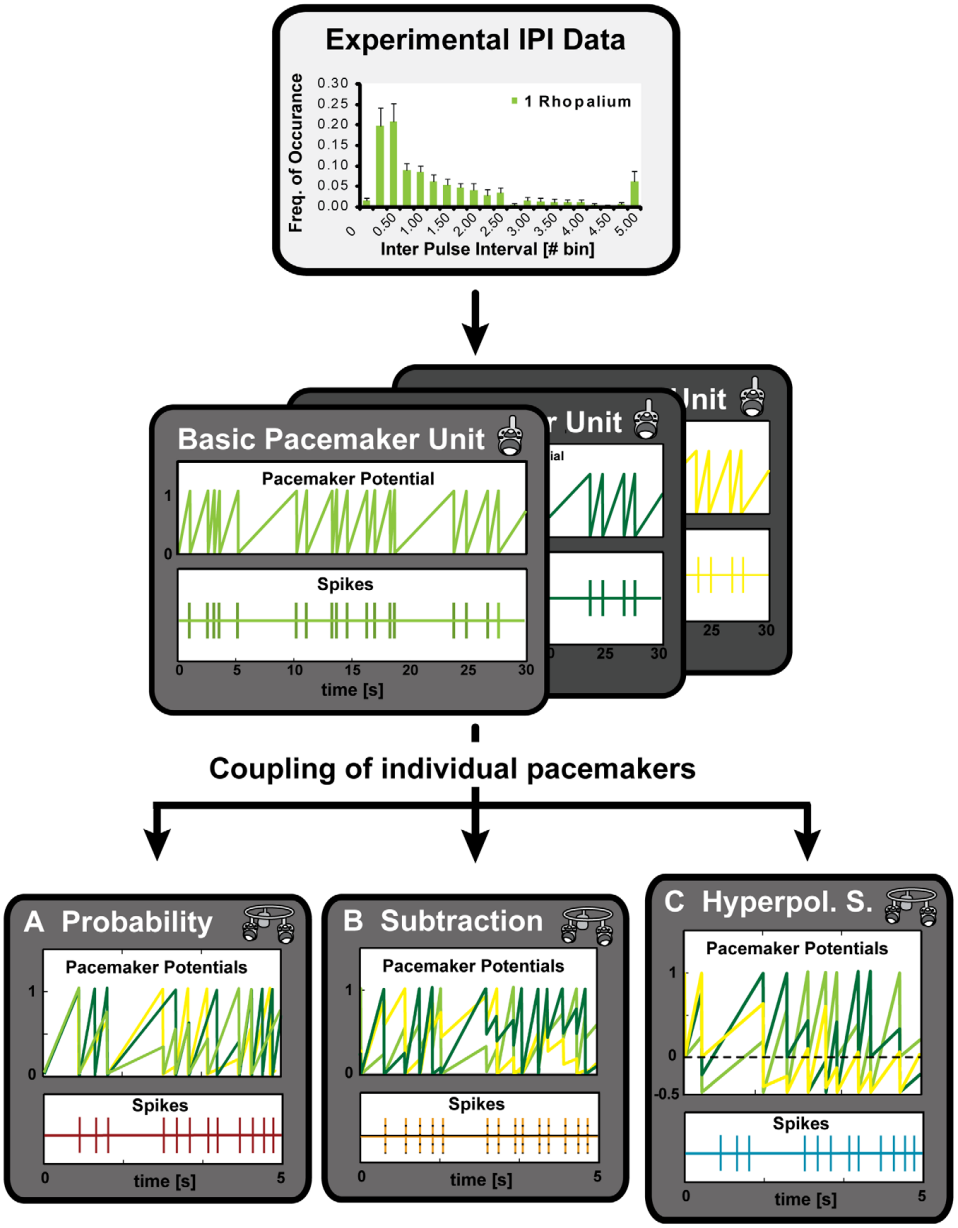
Three different strategies of pacemaker interaction were implemented numerically. The core of the model was an adjustable number of basic pacemaker units (three shown) with an oscillating pacemaker potential, which elicited a spike once it reached threshold. The slope of the potential was based on the experimental IPI distributions of animals with one rhopalium in a way that the resulting IPI distribution of a basic pacemaker unit was identical to the experimental one. In order to couple individual pacemakers, three different strategies were implemented; resetting the pacemakers with a certain probability to the baseline potential (A, probability model), subtracting a certain amount of the pacemaker potential maximally down to baseline (B, subtraction model) or to a certain value below baseline (C, hyperpolarizing subtraction model) once one pacemaker potential reached threshold. The resulting IPI distributions were then compared to the experimental data.

The model of pacemaker interaction was programmed using MATLAB software. It generated swim pulses for a system of coupled CPGs, based on the experimental IPI distributions of animals with a single rhopalium (Fig. 2), for each of the different light conditions.

The core of the model was the basic pacemaker unit, consisting of a linearly increasing potential, which generated a spike once reaching threshold. After spiking, the potential was reset to baseline. The rising slope of the potential was adjusted to the IPI distributions of animals with one rhopalium in a way that a single basic pacemaker unit replicated these distributions upon a sufficiently high number of model runs (Fig. S1). A time point in the model corresponded to 10 ms, and one model run to 30 s, corresponding to the length of the experimental recordings.

The following three strategies were implemented to couple multiple basic pacemaker units. The pacemaker reaching threshold first would cause a swim pulse and interact with the other pacemakers either (i) by resetting them to baseline with a certain probability (probability model, Fig.2, B), (ii) by subtracting a certain amount from the pacemaker potential of the other three rhopalia, with the pacemaker potential decreasing maximally to baseline (subtraction model, Fig.2, B), or (iii) to a certain value below the baseline of resetting (hyperpolarizing subtraction model, Fig.2, C). A coupling strength of 0 percent refers to an independent operation of the pacemakers, while a coupling strength off 100 percent implies fully resetting links between pacemakers.

In order to account for the travel time of spikes across the nerve net of the bell, which was estimated to be 25 ms at maximum according to travel speeds of potentials in box jellyfish nerve cells [24], all spikes generated within this interval were counted as one.

The numerical model results were obtained by simulating 30 s (one run) of pacemaker interaction 200 times. At this number of repetitions, the coefficient of variation of successive runs of the model was reduced to 0.5 percent. The model output was compared to the experimental data of animals with four rhopalia by two different methods, the Kullback-Leibler-Divergence and the Sum-of-Mean-Squares. Additionally, both methods were applied with a restriction, excluding all model outcomes from the evaluation for which the proportion of IPIs shorter than 250 ms exceeded 5 percent. This restriction was applied in order to account for the fact that the small proportion of IPIs shorter than 250 ms (<5 percent) was a typical feature of all experimental IPI distributions. A statistical analysis of the difference between mean, median and standard deviation of the model IPIs versus the experimental IPIs was used as an additional measure for comparison of key features of the model and experimental distributions.

## Results

### Swim pulse analysis of animals with one and four rhopalia under different light conditions

**Figure 3:**
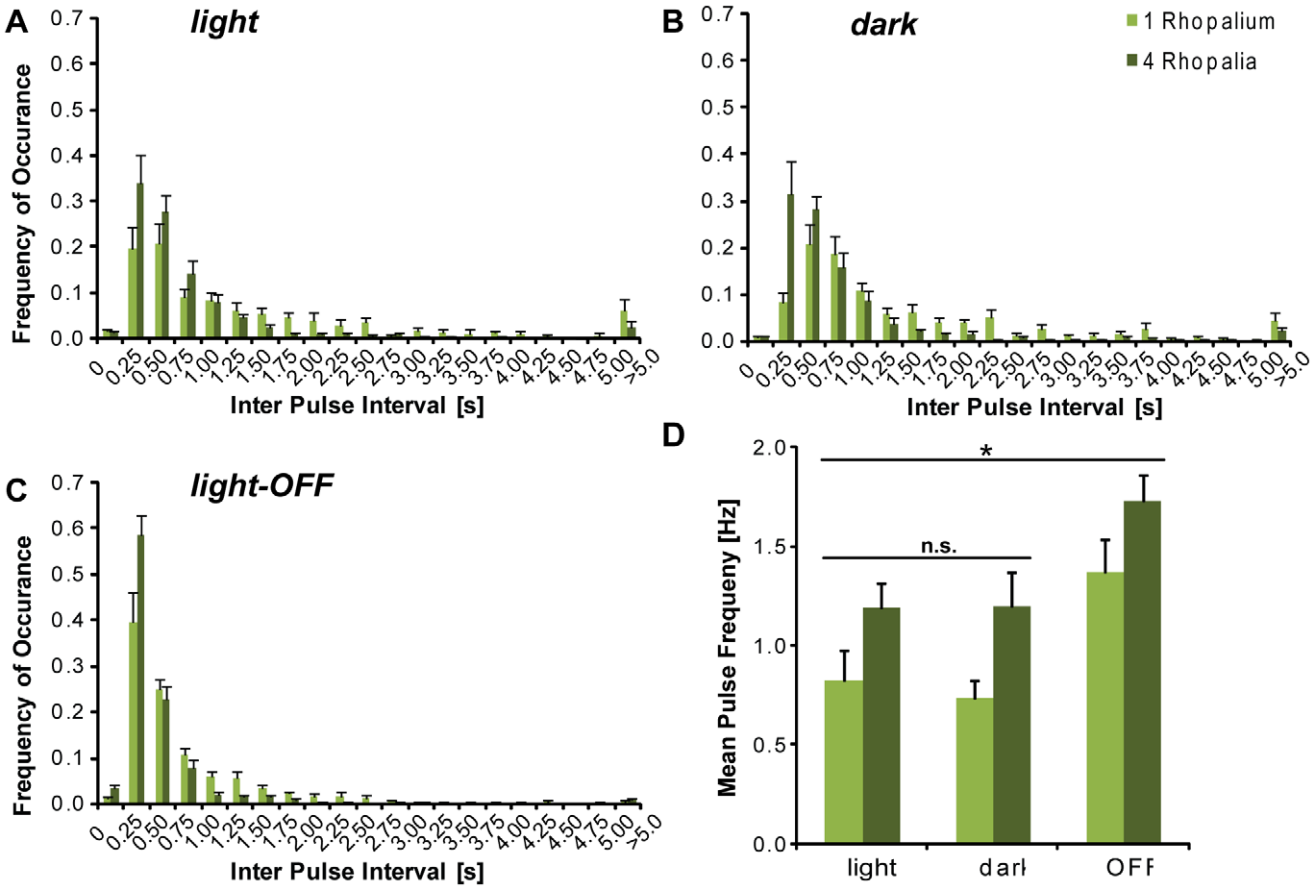
Swim pulse characteristics of animals across light conditions and for different numbers of rhopalia. Panels A, B and C show IPIs of animals in the setup during the *light* (A), *dark* (B) and *light-OFF*(C) condition. The experiments were conducted using intact animals with four rhopalia (dark green) and animals with one rhopalium (light green). For each light condition, the IPI distribution of animals with four rhopalia was shifted towards shorter intervals, as compared to animals with one rhopalium. The mean pulse frequencies (D) for the constant light conditions (*light* and *dark*) were not significantly different for both rhopalial conditions, while the *light-OFF* condition resulted in a significantly increased pulse rate (n = 10, t-test. Values are presented as mean ± S.E.M.

The IPI distributions of *T. cystophora* medusae obtained by high-speed camera observation of tethered swimming animals were in agreement with observations from several previous studies of box jellyfish: They had characteristic long tails towards longer IPIs, which has been described for electrophysiological recordings from single isolated pacemakers [16]–[17] and intact animals [26]. Decreasing the number of rhopalia from four to one decreased the mean and median pulse frequency significantly (p<0.01, n = 10, all tests: one-way ANOVA; followed by Turkey Kramer), while the increase in standard deviation was not significant (Fig. 3, Table S1) [26].

The visual behavior we observed was in accordance with previous electrophysiological and behavioral observations of single pacemakers and intact animals [16]. The mean pulse frequency of animals with four rhopalia was not significantly different (p>0.1) between the *light* condition with 1.19 Hz±0.126 Hz (n = 10, all values mean ± SEM) and *dark* condition with 1.20 Hz±0.175 Hz (n = 11), respectively. It increased significantly for *light-OFF* to 1.73 Hz±0.129 Hz (n = 10, p<0.009) and decreased with less significance for *light-ON* to 0.89±0.13 Hz (n = 5, p<0.1). Similarly, for animals with one rhopalium, the mean pulse frequency of the constant light conditions was not significantly different, but increased significantly for *light-OFF* to 1.37 Hz±0.16 Hz (n = 10, p<0.02) and decreased for the *light-ON* condition to 0.52 Hz±0.1 Hz (n = 4, p<0.01). Corresponding to the mean, the median pulse frequency was significantly different for the *light-OFF* as compared to constant light conditions, while the standard deviations did not differ significantly (Fig. 3, Table S1).

Importantly, less than 5 percent of the IPIs in all experimental conditions were shorter than 250 ms. In all recordings, no IPI shorter than 200 ms was observed, which corresponded to the average contraction time for *T. cystophora* [30].

### Qualitative Analysis of the different modes of coupling in the numerical model

**Figure 4:**
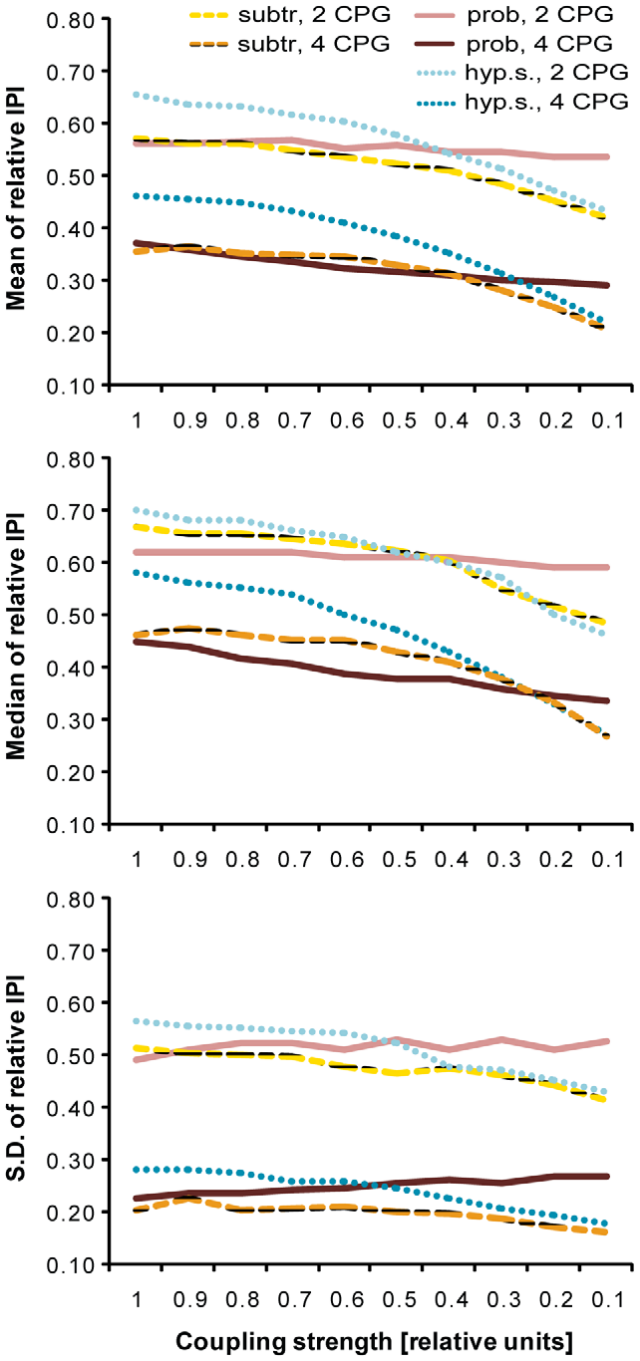
Qualitative analysis of the numerical model of pacemaker interactions for the *light* condition. We analyzed the mean (A), median (B) and standard deviation (C) of the IPIs of the models with different coupling strength and numbers of pacemakers. All values are relative to the values of a single basic pacemaker unit. For all models, the mean and median IPI, as well as the standard deviation decreased with an increasing number of pacemakers. While the mean IPI of the probability model decreased for decreasing coupling strength in a linear way, the IPI of the subtraction model decreased in a way best fitted by a second order polynomial. The dynamics of the subtraction model mean that coupling of 60 percent yielded the same values as full coupling of 100 percent, and could therefore be considered fully resetting. The hyperpolarizing subtraction model had similar dynamics and similar values for low coupling strength as the subtraction model, but higher initial values for fully resetting coupling, as well as a steeper slope of the dynamics.

A single basic pacemaker unit of the model reproduced the IPI distribution of animals with a single rhopalium accurately (Fig. S1, note that the basic pacemaker unit was the same for all types of models). As has been described before for models based on electrophysiological data [26], the median IPI, as well as the mean IPI produced by all models decreased for an increasing number of CPGs, as did the standard deviation of IPIs (Fig. 4). The correlation between coupling strength and median IPI as well as mean IPI, respectively, was linear for the probability model, while it was non-linear for both subtraction models, best fitted by a second order polynomial. Therefore, even for coupling strength of 60 percent, the subtraction models effectively behave in a fully resetting way (Fig. 4, A, B). For the hyperpolarizing subtraction model, the non-linear relation had a substantially steeper slope and a higher initial value at full coupling than the other two models, leading to increased mean IPIs for stronger coupling (Fig.4, A). There was a very weak negative correlation between the standard deviation of the IPI distribution and increasing coupling strength for the subtraction model, while this correlation was stronger and positive for the probability model (Fig.4, C).

### The probability and subtraction model interaction of two pacemakers fit the experimental observations best

The probability and subtraction model were run with coupling strengths from independent to fully resetting as well as two, three or four pacemaker units, in the *light* and *light-OFF* condition, respectively. The best fitting coupling strength for each number of CPGs, as well as the best fitting number of CPGs was evaluated by comparing the model to the experimental IPI distributions of animals with four rhopalia, using both the Sum-of-Mean-Squares and the Kullback-Leibler-Divergence as means of comparison. Both methods selected the same coupling strengths and numbers of pacemakers as best fit for the respective light conditions. Excluding all model results which produced a larger than 5 percent proportion of IPIs shorter than 250 ms confirmed the results of the model evaluations obtained without this constraint.

**Figure 5:**
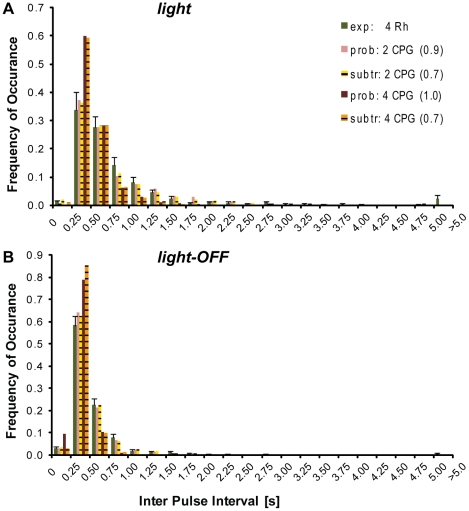
The subtraction and probability model interactions of two pacemakers accurately reproduced the experimental IPI distributions. The experimental IPI distributions of animals with four rhopalia (dark green) for *light* (A) and *light-OFF* (B) are shown together with the results of the probability and subtraction model for two and four CPGs. All model results were obtained by using the optimal coupling (in brackets, 0 = independent, 1 = full strength), and were evaluated by comparing the model IPI distributions to the experimental ones. The model IPIs resulting from the interaction of two pacemakers (light red, probability model, light purple, subtraction model) neatly fitted the experimental data of animals with four rhopalia. The model interactions of four pacemakers did not reproduce the experimental data as adequately. The distribution for both models was shifted to shorter IPIs for four CPGs (dark red, probability, dark purple, subtraction). Experimental values are presented as mean ± S.E.M.

The optimal coupling strength of four CPGs was fully resetting for both light conditions and both the probability and subtraction model (Fig. 5). Recall that, although the optimal coupling strength of the subtraction was not 100 percent, the subtraction model effectively behaved like a fully resetting network for coupling strengths down to 60 percent (Fig. 4). In the case of two coupled CPGs, a fully resetting coupling was optimal for the *light* and *light-OFF* condition for both the probability and subtraction model.

Given optimal coupling strength, the probability and subtraction model with two pacemakers fit the experimental data better than with four pacemakers (Fig. 5). A statistical analysis of the difference between mean, median and standard deviation of the model IPIS versus the experimental IPIs was used as a measure for the similarity between model and experimental distributions. Using this statistical approach, the results from the Sum-of-Mean-Square and Kullback-Leibler-Divergence comparison could be confirmed. The difference between both the probability and subtraction model with four CPGs and the experimental values in the mean and median IPIs, and the IPI standard deviation was highly significant (P<0.01, Kruskal-Wallis Test, followed by Dunn's Multiple Comparisons Test, Table 1). For the models of two CPGs with optimal coupling, none of the three features differed significantly from the experimental data, indicating that this condition adequately described the coupling of the CPG system. The probability and subtraction model did not differ significantly in any of the three features for the same number of CPGs.

**Table 1:** Statistical evaluation of the numerical models.

Nr. of CPGs,	mean	median	s.d.
model (coupling)			
**2, prob (0.9)**	n.s.	n.s.	n.s.
**2, subtr (0.7)**	n.s.	n.s.	n.s.
**4, prob (1.0)**	***	**	***
**4, subtr (0.7)**	***	**	***
**4, subtr (1,** −**0.425)**	n.s.	n.s.	***

Statistical evaluation (Kruskal-Wallis Test, followed by Dunn's Multiple Comparisons Test, * P<0.05, ** P<0.01, *** P<0.001) of the mean, median and standard deviation (s.d.) of the IPIs generated by the numerical models compared to the experimental data for the *light* condition Is shown. The lack of statistically significant difference between the model and experimental values was taken as a measure for the ability of the respective models to properly capture the features of the animal IPI distributions. The high and very high significance of the difference between the subtraction and probability model interactions of four CPGs to the experimental data clearly showed the inability of these models to describe the coupling of the pacemakers of *T. cystophora* adequately. Both types of models with two CPGs produced mean, median and standard deviations which were not significantly different from the experimental data. The hyperpolarizing subtraction model interaction of four pacemakers did so for the mean and median IPIs, while the standard deviation of the IPIs differed in a highly significant way.

### The hyperpolarizing subtraction model produced interactions of four pacemakers that adequately fit the experimental data

**Figure 6:**
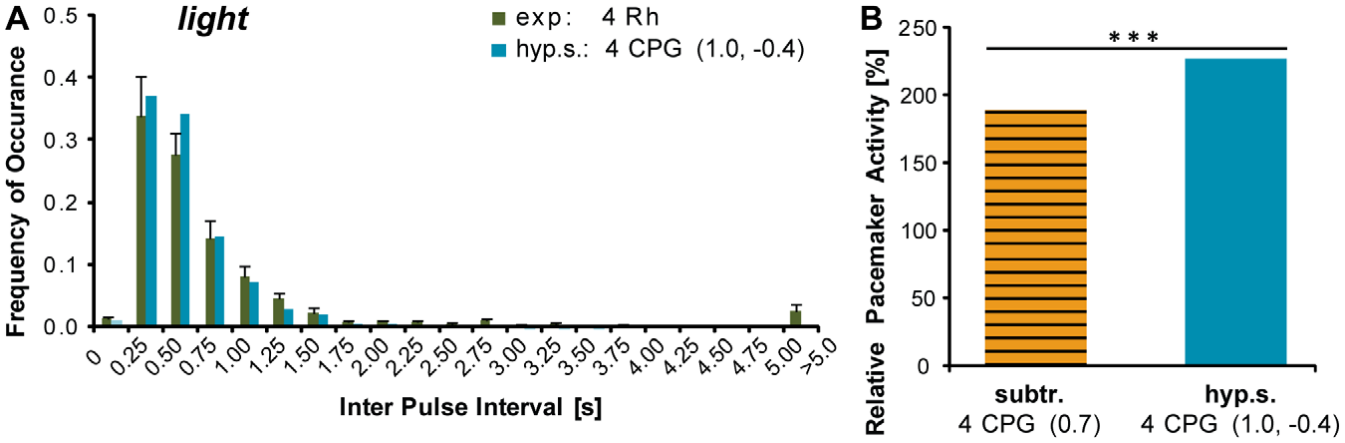
The hyperpolarizing subtraction model with four pacemakers fitted the experimental data adequately and increased the dominance of individual pacemakers. The hyperpolarizing subtraction model with a fully resetting coupling and a lower threshold of −42.5 percent of the baseline was able to closely reproduce the experimental IPI distributions (A, data shown for the *light* condition). Hyperpolarizing links lead to a stronger dominance of the most active pacemaker. Panel B shows the relative activity of one pacemaker stimulated by *light-OFF* while the other three pacemakers where driven by the *light* condition, for the simple and the hyperpolarizing subtraction model. The proportion of spikes elicited by the *light-OFF* stimulated pacemaker was significantly higher for the hyperpolarizing subtraction model. Experimental values are presented as means ± S.E.M.

In the case of the hyperpolarizing subtraction model, the potential of a basic pacemaker unit was free to decrease below the baseline potential (Fig. 2, C). This assumption generated a resetting behavior with an increased average time to spike as compared to the other two models. Using the hyperpolarizing subtraction coupling, the interaction of four pacemakers was able to closely reproduce the experimental IPI distribution (Fig. 6, A). Mean and median IPIs of the model did not differ significantly from the experimental values (Table 1, p>0.05, Kruskal-Wallis Test, followed by Dunn's Multiple Comparisons Test). The standard deviation of this model was significantly smaller than the experimental one, indicating also this model was not able to correctly reproduce the proportion of long intervals.

Furthermore, the hyperpolarizing subtraction model showed a stronger dominance of the most active pacemaker over the whole system than the simple subtraction model. If one CPG was driven by a *light-OFF* stimulation, while the other three responded to constant *light* stimulation, the proportion of spikes elicited by the *light-OFF* activated pacemaker as compared to the other pacemakers was significantly higher for the hyperpolarizing than for the simple subtraction model (Fig 6 B).

### The proportion of long interpulse intervals of the experimental data was not captured by any of the model interactions

**Figure 7:**
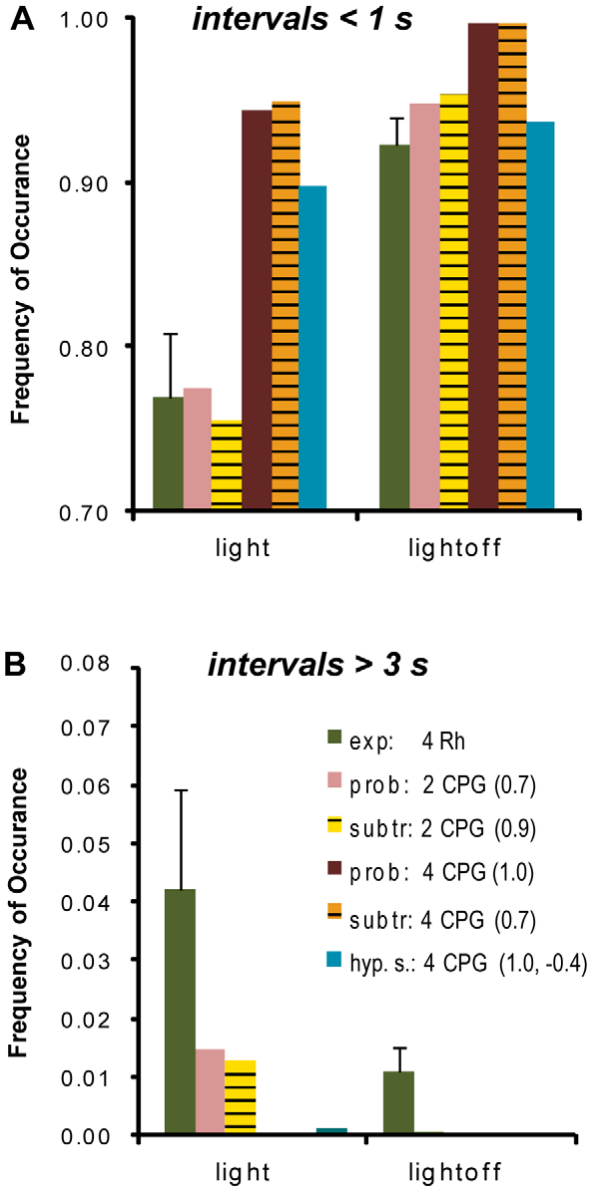
The model interactions of two and four pacemakers did not account for the proportion of long intervals in the experimental data. The proportion of short IPIs (<1 s, A) of intact animals (dark green) was well captured by the model interactions of two CPGs in case of the probability and subtraction model. The probability and subtraction model interactions of four CPGs resulted in a proportion of short intervals distinctly larger than the one of the experimental data. Neither the model interactions of two nor of four CPGs of any mode of coupling could adequately reproduce the proportion of long intervals of the experimental data (>3 s, B). The probability and subtraction model interaction of four pacemakers did not result in any long intervals at all. The proportion for the interaction of two pacemakers in the case of the probability and subtraction model, as well as for the interaction of four pacemakers in the case of the hyperpolarizing subtraction model was multiple times smaller than the proportion of the experimental data. Experimental values are presented as means ± S.E.M.

Although the best fitting models captured most of the features of the experimental IPI distributions, they did not reproduce the proportion of long intervals (>3000 ms) of the experimental data (frequency of occurrence in *light*: 0.0422±0.0170 S.E.M., *light-OFF*: 0.0110±0.0039 S.E.M., Fig. 7). No coupling strategy of four pacemakers did produce any long intervals for the *light-OFF* condition, while the hyperpolarizing subtraction model was the only model of four pacemakers that produced a very small proportion of long intervals for the *light* condition. Even for the best fitting models of two CPGs, the proportion of long intervals in the experimental data exceeded the proportion in the model data by more than five times. On the contrary, the model interactions of two pacemakers neatly reproduced the proportion of short intervals (<1000 ms). As shown before, the simple subtraction and probability-coupling interaction of four rhopalia did not reproduce the key features of the models well, and substantially overestimated the number of short intervals.

## Discussion

### Swim pulse analysis of animals with one and four rhopalia under different light conditions

The behavioral swim pulse data for *T. cystophora* was in agreement with observations from earlier studies of box jellyfish behavior [16] and electrophysiology [26]. The mean pulse frequency of both rhopalial conditions increased significantly for *light-OFF* and decreased for *light-ON*, while there was no significant difference for the constant light conditions, which has also been observed in electrophysiological studies of isolated pacemakers [16], [17]. The fact that only a proportion of less than 5 percent of swim IPIs was shorter than 250 ms in all our observations and no IPI was shorter than 200 ms corresponds neatly to the mean pulse duration of 200 ms described for *T. cystophora* [30], which might be dictated by the mechanics of the bell. Restrictions imposed by bell mechanics were shown in a hydrozoan jellyfish, which also has jet propulsion swimming [31].

Despite the accordance of our observations with earlier studies as far as general trends are concerned, the pulse frequency we described was higher in absolute values as compared to data of animals freely swimming in the mangrove swamps [16]. This could be explained by the fact that the light conditions in the mangroves differ from the very simple and controlled light conditions in our experiments, and therefore also trigger different pulse responses. It might also be that the difference in pulse rate was a consequence of the size of the experimental animals used. The animals observed in the mangroves were nine millimeters in diameter on average [16], while in our study the average diameter was four millimeters. It has been described for other species of jellyfish that the pulse frequency is correlated to the size of the medusae with an inverse relation [32]. For *T. cystophora*, however, a similar relation is not described. Moreover, there was no significant correlation between size and mean IPI observed in our experiments, taking into account animals from 2.5 cm to 5 cm in bell diameter (Pearson Correlation, P>0.05, for all rhopalial and light conditions, Fig S2).

Another argument for the difference in pulse frequency could be that tethering the animals had an effect on their behavior. However, the fact that the response to the different light conditions in our experiments, as well as the shape of the IPI distributions, was in accordance with previous observations [16], [17], [24], [26], speaks against an atypical behavior of the medusae. Moreover, their tentacles were extended during experiments and their pulsing occasionally paused for intervals of several seconds. Stressed animals usually swim with continuous fast pulses with their tentacles retracted.

The IPI distributions of animals with one rhopalium differed from the electrophysiological ISI distributions of isolated pacemakers, which have distinctly longer mean and median IPIs [16]. One reason for this difference might be that the function of isolated pacemakers is affected by the lack of feedback from the whole nervous and body system, which leads to a decreased pacemaker frequency.

### Biological Interpretation of the Model

**Figure 8:**
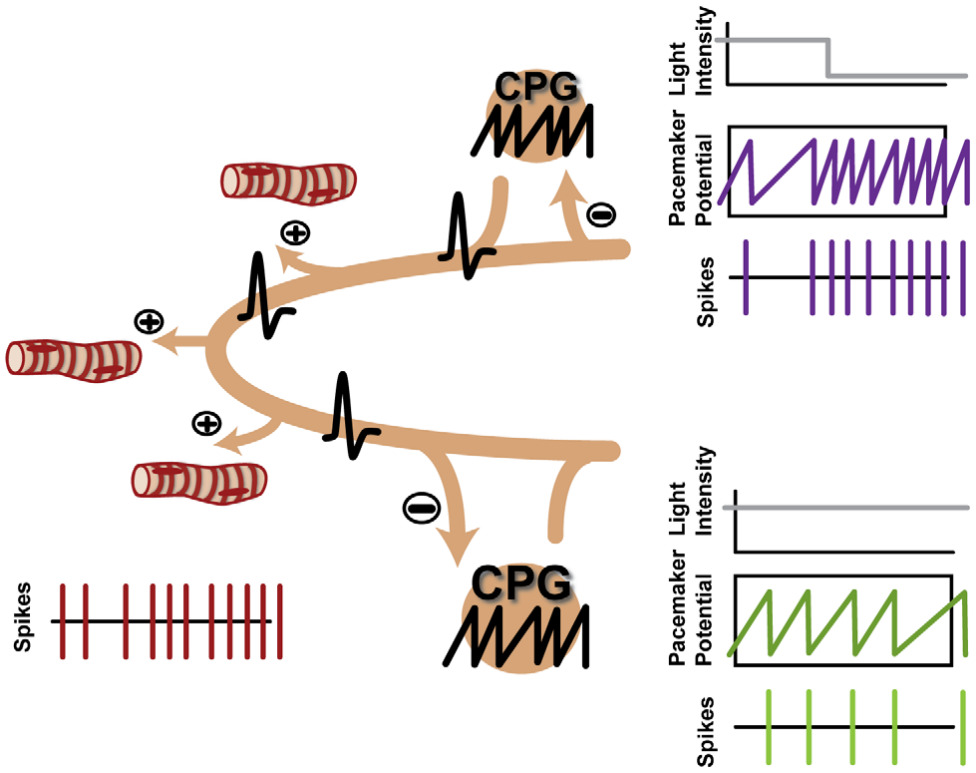
A functional suggestions for pacemaker coupling. In a basic pacemaker unit (CPG) a potential oscillates from baseline to threshold, with its frequency depending on the light condition. When the potential reaches threshold, a spike is generated and transmitted to the nervous system of the jellyfish. Spikes generated by the CPGs are transmitted to the muscles by the ring nerve and nerve net and translated into contractions in a one-to-one manner. The pacemakers are mutually connected in an inhibitory way, transforming the spikes generated by other pacemakers into hyperpolarizing potentials, which decrease the pacemaker potentials according to the subtraction or hyperpolarizing subtraction model, respectively. By this mechanism, the spike frequency of a pacemaker is reduced if another pacemaker increases its firing frequency. The overall swim speed of the jellyfish results from the pooled action of the four CPGs.

The differences between the probability and subtraction model, given optimal coupling strength, were not statistically significant (p>0.05, Kruskal-Wallis Test, followed by Dunn's Multiple Comparisons Test). In terms of generating the relevant IPIs, both models performed equally well. Intuitively, the subtraction mode of coupling can more straightforwardly be translated into biological correlates (Fig. 8): The spikes of the basic pacemaker units in each rhopalium travel around the bell via the ring nerve and nerve net and elicit muscle contractions in a one to one manner. Possibly, the connections between ring nerve and rhopalia are not only outgoing into the nerve net of the bell, but also incoming from the ring nerve into the rhopalial neuropil. Anatomic observations describe two parts of the ring nerve that branch off the main nerve bundle to enter the stalk and the rhopalia. They have been interpreted as connections between adjacent rhopalia, which allow for integration of information between the rhopalia [33]. There are hints that the activity of one pacemaker suppresses the activity of the other pacemakers [24]. Both excitatory and inhibitory neurotransmitters have been described for other jellyfish species [34]. Therefore it is conceivable that the connections from neurons of the ring nerve to the pacemaker neurons are inhibitory, and with every spike elicited by one CPG, the other CPGs could receive hyperpolarizing inputs that decrease their membrane potentials and increase the time to the next spike. Due to the bipolarity of the nerve net of box jellyfish, the impulses can only travel around half the bell before they cancel out by running into each other and further transmission is blocked by the refractory period of the nerve fibers [24]. This way, the pacemaker which elicited a spike is not affected by any hyperpolarizing input, and its potential is reset to baseline of its regular oscillation. Depending on the strength of the inhibitory connections, different coupling strengths are possible, from only weak coupling to fully resetting and to hyperpolarizing resetting.

### The probability and subtraction model interaction of two pacemakers provide the best fit to experimental observations

For none of the light conditions did the interaction of four CPGs in the probability and subtraction model provide a close fit of the experimental data. But coupling only two CPGs to a fully resetting network produced IPIs whose characteristics did not differ significantly from the experimental ones (Table 1, Fig. 5). Both for two and four coupled CPGs, fully resetting coupling was the best fit for the *light* and *light-OFF* condition for the probability as well as for the subtraction model (Fig. 5).

Fully resetting links were already proposed in earlier studies [22], [25]. A more recent study [26] suggests that semi-independent coupling, rather than fully resetting links, would generate directional swimming. However, there is evidence for different species of cubomedusae that steering might be conducted by the differential contraction of structures shaping the water expulsion opening of the medusa [35] [30].

Interestingly, the fact that the coupling of four CPGs using the probability and subtraction model was not able to account for the observed IPI distribution of animals with four rhopalia, has been described before by Satterlie and Nolen [26]. The model in this previous study was mechanistically similar to our probability model and used fully resetting and independent coupling. The results also agree with ours in that the model interactions of two pacemakers, but not of four, produced the closest fit to the mean IPIs of animals with four rhopalia.

There are several possibilities why the average activity of two CPGs provided such good fit for the swim pulse data of box jellyfish medusae. First, there might actually be only two CPGs active at a time, while the other two are silenced by some autonomous mechanism. Evidence for this might be the observation that pacemaker signals from isolated rhopalia show periods of bursting activity which alternate with pauses of several tens of seconds [17]. Against this hypothesis speaks the fact no animal with one rhopalium showed comparably long intervals of immobility in our experiments. A second hypothesis, which is in conformity with our observations, assumes an external control that silences two pacemakers in random fashion, but for some reason is not active if only one rhopalium is left. However, this is a purely theoretical concept, and we cannot see any biological relevance for having four pacemakers and randomly silencing two of them.

### The hyperpolarizing subtraction model produced an adequate data fit with four pacemakers interacting

The third and biologically more plausible hypothesis to explain why the interactions of four pacemakers in the subtraction and probability model did not reproduce the experimental data well, is to assume that they did not capture all aspects of coupling between the pacemakers in box jellyfish medusae. We therefore implemented a further expanded mode of coupling in the hyperpolarizing subtraction model (Fig. 2, C). This model allowed the potential of a basic pacemaker unit to decrease below the baseline of resetting, by subtraction caused by spiking action of another pacemaker. It therefore increased the time to spike as compared to the probability and subtraction model. The hyperpolarizing subtraction model was able to closely reproduce the experimental IPI distribution (Fig. 6, A). Only the proportion of long IPIs and correspondingly the standard deviation of the experimental data were still underestimated by this type of coupling (Fig. 7).

A functional advantage of the hyperpolarizing resetting model was that individual pacemakers gained a larger impact on the whole system. One pacemaker with a higher pulse frequency than the other pacemakers dominated the system by reducing the chance of any other pacemaker to reach threshold due to the hyperpolarizing inputs. This mechanism only works if the dominating pacemaker increases firing frequency, similar to our simulation, in which one pacemaker reacted to *light-OFF*, while the others continued in the constant *light* mode (Fig 6, B). A pacemaker decreasing its frequency in a *light-ON* reaction would not become dominant. This disparity corresponds to the difference in acute relevance of the *light-OFF* and *light-ON* situation to box jellyfish medusa. A sudden decrease in light intensity is an indicator for a potential threat to the medusae, such as mangrove roots which can potentially harm the fragile body, or areas outside the light shafts, where they will not find food. With hyperpolarizing links, the system could effectively increase the swim speed of the animal to swim away from the potential threat, even if only one eye is stimulated.

### The proportion of long and short IPIs in the experimental IPI distributions was not reproduced by any of the models

Although the best fitting models – two CPGs or four CPGs with hyperpolarizing links, respectively - captured most of the features of the IPI distributions, they did substantially underestimate the proportion of long intervals (>3000 ms) observed in the experiments. Interestingly, a similar result has been described before [26].

This finding strongly suggests additional mechanisms to pacemaker signals that control the swim speed of *T. cystophora* medusae. A putative source for such control is the gastrointestinal system, which might have a calming effect on medusa swim pulsing in order for the food to be processed and the manubrium (mouth) to maneuver. Anatomical observations might support this hypothesis: a gastrodermal nerve has been described to enter the rhopalia of box jellyfish. However, its origins have not yet been discovered [33]. It has been observed in a hydrozoan species that the stimulation of the radial musculature of the gastrointestinal system slowed down and compromised the regularity of the swimming contractions [36]. The fact that the proportion of long IPIs was reduced upon *light-OFF* stimulation speaks in favor for the hypothesis of additional control. The proportion of long IPIs immediately after *light-OFF* stimulation was even smaller than the values in Fig. 7, because the swim pulse frequency was at its maximum for ten seconds after *light-OFF* stimulation, before the pulse rate declined again. This has been described for isolated pacemakers as well [16]. As discussed before, for *T. cystophora*, a sudden drop in light intensity indicates a situation that requires action. Therefore, if faced with such a condition, the additional regulation of swimming by another system than the visual should be suppressed.

### Conclusion

Our results support early models of the box jellyfish pacemaker system, which propose fully resetting links between the individual pacemaker centers [22], [25]. However, studying the system not in terms of isolated pacemakers, but in its bodily environment, we made some unexpected findings which indicate that there is more to the system than only resetting links between for pacemaker centers. Our data supports the idea of hyperpolarizing links between the pacemakers, increasing the impact of individual pacemakers, especially in situations which indicate danger to the animal, while keeping the regularity and reliability of a multi-pacemaker system. Moreover, we found evidence for an additional mechanism, which slows down the swim pulse frequency and produces long IPIs, which do not result from a simple interaction of the four CPGs. Our results therefore provide guidelines for future research on the physiological links of cubozoan CPGs. Moreover, our results from a cnidarian system support the conclusions from recent work of pacemaker research in bilaterians, which show that CPGs have to be studied in their bodily and nervous environment in order to fully understand their characteristics and function [5], [6].

## Supporting Information

Figure S1 IPIs of animals with one rhopalium and simulation of one basic pacemaker unit. For the basic pacemaker unit of the numerical model we adjusted the slope of the oscillating potential to the IPI distribution of animals with one rhopalium. The basic pacemaker unit was the same for all models. It reproduced the experimental IPI distributions neatly. Experimental values are presented as means ± S.E.M.
(TIF)

Figure S2 Bell diameter and mean IPI were not correlated. The bell diameter of experimental animals was between 2.5 and 5 mm. There was no significant correlation between the size of the animals and their mean IPIs for the different light conditions and the one (A) or four (B) rhopalia conditions (Spearman Correlation, P: significance of correlation factor being different from zero).
(TIF)

Table S1 Comparison of IPI characteristics of animals with one and four rhopalia for different light conditions. ‡ ANOVA followed by Tukey-Kramer Test, † unpaired t-test, ^+^ Kruskal-Wallis followed by Dunn's Multiple Comparisons Test, ^−^ Mann-Whitney Test. The mean and median pulse frequency of animals with four rhopalia were significantly to animals with one rhopalium, while the decrease of the standard deviation was not significant. For both rhopalial conditions the standard deviation of the light conditions did not differ significantly. The difference between the mean and median pulse frequency of the constant light conditions was not significant either, while the light-OFF condition differed significantly from the constant light conditions for animals with both one and four rhopalia. Values are presented as means ± S.E.M.
(DOC)
