# Supplementary figures and images for: Setting the Pace: New Insights into Central Pattern Generator Interactions in Box Jellyfish Swimming
Source: PLoS One. 2011 Nov 2;6(11):e27201. doi: 10.1371/journal.pone.0027201 (PMC3206948; doi:10.1371/journal.pone.0027201)

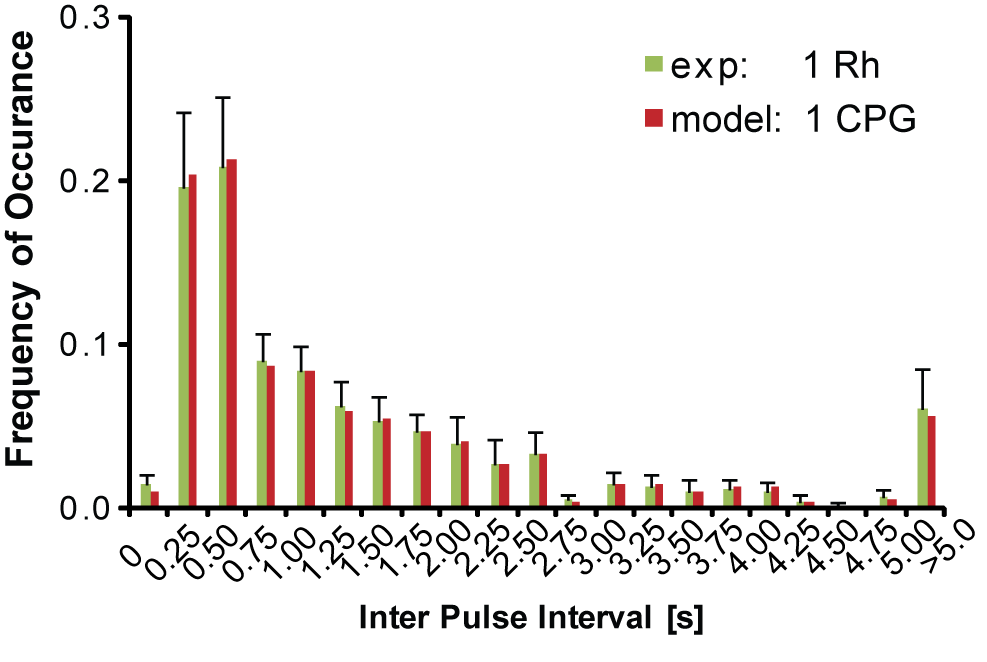

Supplement: Figure S1 — IPIs of animals with one rhopalium and simulation of one basic pacemaker unit. For the basic pacemaker unit of the numerical model we adjusted the slope of the oscillating potential to the IPI distribution of animals with one rhopalium. The basic pacemaker unit was the same for all models. It reproduced the experimental IPI distributions neatly. Experimental values are presented as means ± S.E.M. (TIF) [file pone.0027201.s001.tif]

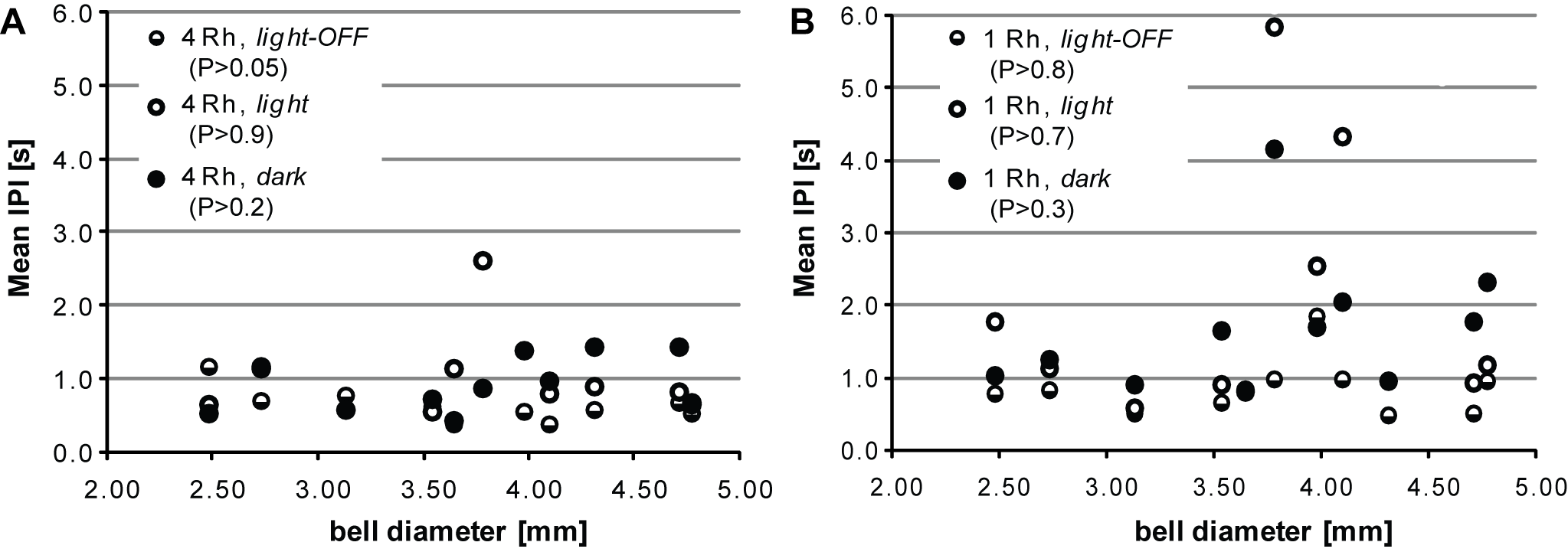

Supplement: Figure S2 — Bell diameter and mean IPI were not correlated. The bell diameter of experimental animals was between 2.5 and 5 mm. There was no significant correlation between the size of the animals and their mean IPIs for the different light conditions and the one (A) or four (B) rhopalia conditions (Spearman Correlation, P: significance of correlation factor being different from zero). (TIF) [file pone.0027201.s002.tif]
