# Supplementary material for: Setting the Pace: New Insights into Central Pattern Generator Interactions in Box Jellyfish Swimming
Source: PLoS One. 2011 Nov 2;6(11):e27201. doi: 10.1371/journal.pone.0027201 (PMC3206948; doi:10.1371/journal.pone.0027201)
Supplement: Table S1 — Comparison of IPI characteristics of animals with one and four rhopalia for different light conditions. ‡ ANOVA followed by Tukey-Kramer Test, † unpaired t-test, + Kruskal-Wallis followed by Dunn's Multiple Comparisons Test, − Mann-Whitney Test. The mean and median pulse frequency of animals with four rhopalia were significantly to animals with one rhopalium, while the decrease of the standard deviation was not significant. For both rhopalial conditions the standard deviation of the light conditions did not differ significantly. The difference between the mean and median pulse frequency of the constant light conditions was not significant either, while the light-OFF condition differed significantly from the constant light conditions for animals with both one and four rhopalia. Values are presented as means ± S.E.M. (DOC) [file pone.0027201.s003.doc]

| **Nr. of Rhopalia** | **light condition** | **mean [Hz]** | | **median [Hz]** | | **s.d. [s]** | |
| --- | --- | --- | --- | --- | --- | --- | --- |
|  |  |  |  |  |  |  |  |
| **one** | ***dark*** | 0.73 ± 0.09 |  | 0.99 ± 0.12 |  | 1.50 ± 0.11 |  |
|  | ***light*** | 0.83 ± 0.15 | n.s. ‡ | 1.1 ± 0.18 | n.s. ‡ | 2.32 ± 0.27 | n.s. ⁺ |
|  | ***light-OFF*** | 1.37 ± 0.16 | ** ‡ | 1.73 ± 0.18 | ** ‡ | 0.60 ± 0.04 | n.s. ⁺ |
|  | ***all*** | 0.97± 0.02 |  | 1.27± 0.02 |  | 1.47 ± 0.06 |  |
| **four** | ***dark*** | 1.20 ± 0.17 |  | 1.66 ± 0.16 |  | 1.05 ± 0.07 |  |
|  | ***light*** | 1.19 ± 0.13 | n.s. ‡ | 1.74 ± 0.17 | n.s. ‡ | 1.14 ± 0.10 | n.s. ⁺ |
|  | ***light-OFF*** | 1.73 ± 0.13 | * ‡ | 2.24 ± 0.08 | * ‡ | 0.65 ± 0.05 | n.s. ⁺ |
|  | ***all*** | 1.36± 0.02 | ** † | 1.87± 0.02 | *** † | 0.95 ± 0.03 | n.s. ⁻ |
